# Nanobodies; new molecular instruments with special specifications for targeting, diagnosis and treatment of triple-negative breast cancer

**DOI:** 10.1186/s12935-022-02665-0

**Published:** 2022-08-06

**Authors:** Hamid Bakherad, Fahimeh Ghasemi, Maryam Hosseindokht, Hamed Zare

**Affiliations:** 1 Department of Pharmaceutical Biotechnology and Isfahan Pharmaceutical Sciences Research Center, School of Pharmacy and Pharmaceutical Sciences, Isfahan University of Medical Sciences, Isfahan, Iran; 2 Department of Medical Biotechnology, Faculty of Medicine, Birjand University of Medical Sciences, Birjand, Iran; 3 Pharmaceutical Sciences and Cosmetic Products Research Center, Kerman University of Medical Sciences, Kerman, Iran

**Keywords:** Nanobody, VHH, TNBC, Diagnosis, Treatment

## Abstract

Breast cancer is the most common type of cancer in women and the second leading cause of cancer death in female. Triple-negative breast cancer has a more aggressive proliferation and a poorer clinical diagnosis than other breast cancers. The most common treatments for TNBC are chemotherapy, surgical removal, and radiation therapy, which impose many side effects and costs on patients. Nanobodies have superior advantages, which makes them attractive for use in therapeutic agents and diagnostic kits. There are numerous techniques suggested by investigators for early detection of breast cancer. Nevertheless, there are fewer molecular diagnostic methods in the case of TNBC due to the lack of expression of famous breast cancer antigens in TNBC. Although conventional antibodies have a high ability to detect tumor cell markers, their large size, instability, and costly production cause a lot of problems. Since the HER-2 do not express in TNBC diagnosis, the production of nanobodies for the diagnosis and treatment of cancer cells should be performed against other antigens expressed in TNBC. In this review, nanobodies which developed against triple negative breast cancer, were classified based on type of antigen.

## Introduction

According to oncology studies in most countries, breast cancer is the most prevalent cancer in women, and this malignancy is the 2nd leading reason for cancer death in women [1, 2]. The 2021 worldwide cancer information indicated that there were about 2,260,000 women detected with breast cancer, and 684,996 women died of breast cancer [3]. In fact, the prevalence and mortality rate of breast cancer in the study population was 11.7% and 6.9%, respectively [3].

Of the numerous breast cancers, TNBC (triple-negative breast cancer) has a more aggressive proliferation and a poor clinical diagnosis and prognosis [2]. TNBC is distinct from other breast cancers with negative expression of HER2, progesterone, and estrogen [2, 4]. Currently, the most common treatments for TNBC are chemotherapy, surgical removal, and radiation therapy, which impose many side effects and costs on patients [1]. TNBC has features compared to other types of breast cancer that increase the importance of early detection. According to epidemiological data, TNBC is more common in women under the 40 years of age, and the survival time of these patients is shorter than that of other types of breast cancer; 40% of these women die in the first 5 years after diagnosis [4]. About 46% of TNBC patients have distant metastases (brain and viscera), and the median survival after metastasis is 13.3 months. The average recurrence time in TNBC and non-TNBC patients is 51 months and 29 months, respectively [4].

**Fig. 1:**
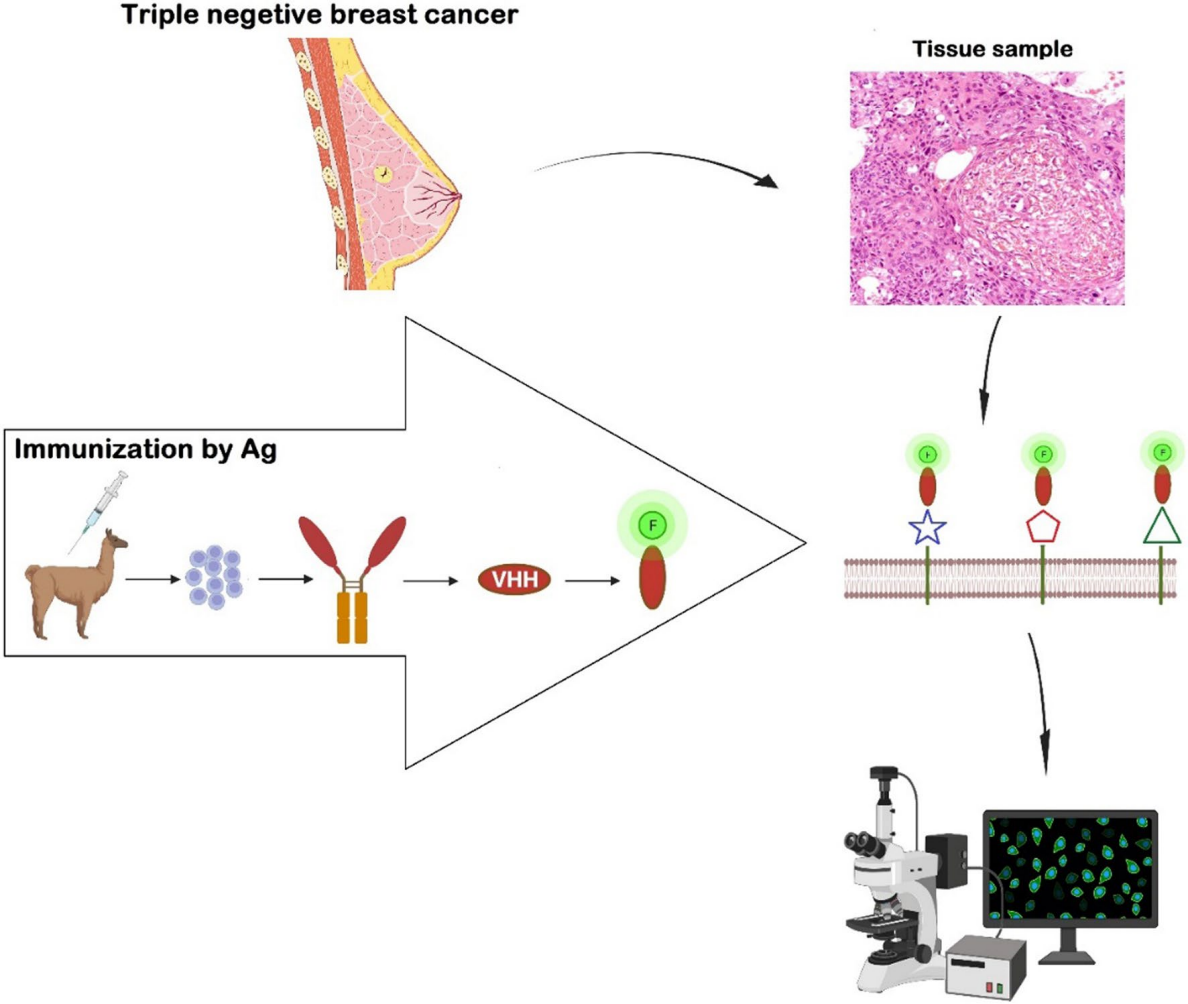
VHH or nanobodies are derived from camel heavy chain antibodies. These nanobodies are labeled by various agents and are able to detect specific antigens in breast cancer tissues

Antibodies, as the most common diagnostic agent, are a great biological tool for diagnosing and targeting cancer (Fig. 1) [5–7]. Although conventional antibodies have a high ability for tumor markers' diagnosis, their large size, instability, and costly production cause a lot of problems [5, 8]. Sharks and camels can manufacture antibodies without light chains, which are called heavy-chain antibodies [9]. The N-terminal part of these antibodies can preserve their antigen-binding ability and can be produced in microorganisms such as *E. coli*, *S. cerevisiae*, *P. pastoris*, and tobacco plants [10]. VHH, or nanobody, is the name of the isolated variable domain of the camelid heavy chain antibody [10]. In fact, in order to differentiate the variable domain of heavy chain antibodies from conventional antibodies, these variable segments are called VHH and VH, respectively. VHHs are the smallest natural antigen-binding fragments and they are known as nanobodies due to their small size (∼2.5 × ∼ 4 nm) [11]. Nanobodies (VHH) derived from camelid heavy chain antibodies have superior benefits, including small size, low-cost manufacture, and good stability, which makes their use in diagnostic kits attractive. Moreover, these nanobodies are able to diagnose mysterious epitopes and haptens that are not available to conventional antibodies [5]. Additionally, VHHs are suitable for use in treatment since they have more than 80% similarity to human VHs [12]. VHHs can be linked (fusion nanobodies) with enzymes, substrates, toxins, radioactive materials, and other biologically active substances to achieve specific functional effects [1]. Nanobodies can achieve certain functional effects by fusing with Fc domains, other nanobodies, and targeted peptides. These hybrid proteins have improved anticancer activity and selectivity [1]. Since HER-2 is not expressed in TNBC diagnosis, the production of nanobodies for the diagnosis and treatment of cancer cells should be performed against other antigens expressed in TNBC such as TNF-α, EGFR, CD3, CTLA-4, STAT3, AKT2, GTP-binding protein Rho, CapG, fibronectin, and CA-IX.

In this article, we summarize and review and nanobodies designed and produced against triple negative breast cancer (TNBC). The use of these nanobodies in designing new diagnostic and treatment methods will be very promising for the effective treatment and early detection of TNBC patients.

### Nanobody or VHH

**Fig. 2:**
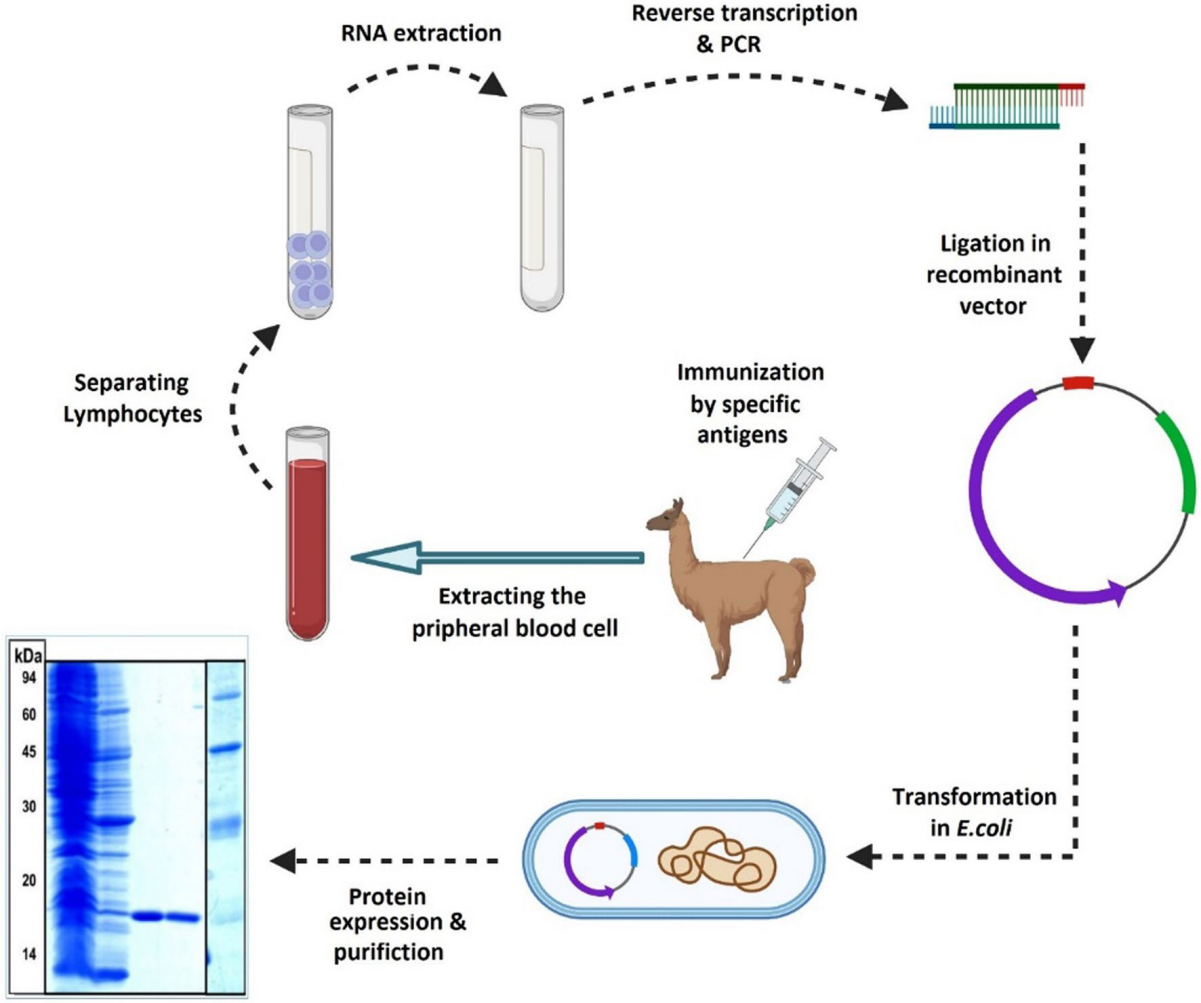
Nanobody production procedure. A camel was immunized with a desired antigen. After lymphocyte separation, mRNA isolation and cDNA synthesis were performed. The variable fragment of heavy-chain antibodies (HCAbs) was amplified by PCR and cloned into the phagemid vector. The variable fragment antibody (VHH) was displayed on the surface of an M13 phage. After panning and selection of colonies with the maximum affinity, soluble VHH was expressed in *E. coli*

By chance, a novel class of antibodies was discovered in camelids in the early 1990s that naturally lack light chains and the first constant domain (CH1) in the heavy chain [9]. VHH, or nanobody, is the N-terminal fragment of this class of antibodies. The antigen binding region of the nanobodies consists of four FRs and three CDRs. The CDR3 domain allows the detection of haptens and cryptic epitopes. These types of epitopes are not detectable by classical antibodies. Manipulation, cloning, and production of nanobodies with high affinity and yields are simply done on microorganisms, animal cells, plant cells, and insect cells due to the lack of a light chain (Fig. 2) [13, 14]. It was shown that the VHHs keep their entire antigen-binding capacity and were regarded as the smallest natural antigen-binding units [9]. The VHH molecule, with a size of less than 15 kDa, 2.5 nm in diameter, and 4 nm in height, was also called nanobody [9]. Significant efforts in nanobody research have been established to provide the technology of production and selection of nanobodies. Some of the companies active in this field are: Camel-IDS, Ablynx, Hybrigenics, Chromotek, etc. [15, 16]. These companies are working to develop therapeutic nanobodies, with 9 currently in clinical trials and more than 15 candidates in the discovery and preclinical stages [17].

### Nanobodies against TNF-α

Among the important factors for the progression of breast cancer, TNFα plays an essential role in promoting the growth of the tumor and metastasis; so that it is proven that anti-TNFα antibodies can prevent the proliferation of breast cancer cells and metastasis [18, 19]. Ji et al. [2] developed a neutralizing nanobody against human TNFα. The TNFα-specific nanobody with a molecular weight of 15 kDa and Kd of 2.05 nM was produced in the *Pichia pastoris* expression system. In their experiment, proliferation of the hTNFα-induced MCF-7 breast cancer cell line was inhibited by TNFα-specific nanobody. Moreover, in a micro-invasion model, the TNFα-specific nanobody inhibited the migration of the MDA-MB-231 and MCF-7 cell lines. In a mouse model experiment, once-daily subcutaneous dosing was performed in the 4 T-1 metastatic breast cancer mouse model. The TNFα level was lower than it was during the earlier stages of tumor formation. Their research emphasized the importance of neutralizing low levels of TNF in the tumor microenvironment in order to sensitize the potential of chemotherapy response for medical use [2].

In another study that was later conducted by Ji et al. [1] a fusion form of the previously developed anti-TNFα nanobody was designed to improve the anti-tumor activity of the TNFα-specific nanobody against TNBC. Using genetic engineering approaches, they developed three RGD4C-fused anti-TNFα nanobody configurations and studied their antitumor activities both in vitro and in vivo. Among three configurations, the fusion nanobody V-L-R-H (VHH-Liker-RGD4C-6xHis) effectively attached to αvβ3 region and inhibited cell proliferation and migration of the MDA-MB-231 cell line. Moreover, this fusion nanobody inhibited the TNFα-mediated PI3K/AKT/NF-κB and integrin αvβ3 focal adhesion kinase signal pathways. They study the therapeutic effect of V-L-R-H in vivo by establishing a xenograft mouse model of MDA-MB-231. Compared to VHH, the V-L-R-H considerably reduced the growth of tumors and lung metastases in mice, and it had no appreciable side effects. Immunofluorescence and immunohistochemistry results revealed that the V-L-R-H could efficiently decrease the TNFα concentration in the tumor microenvironment. Moreover, the expression of HIF-1α and Ki67 was reduced in tumor cells, which caused the tumor's morphology and structure to be destroyed. Finally, neovascularization and EMT of tumor cells were inhibited. In fact, fusion nanobodies effectively improved the antitumor activity of the previously developed anti-TNFα nanobody on triple negative breast cancer [1].

### Nanobodies against EGFR

Today, the use of quantum dots in various fields has been widely used in diagnosis and treatment due to their dual nature. Wang et al. [20] used quantum dot (InP/ZnS QD) as the main core of a nanoparticle due to its lower cytotoxicity than other quantum dot cadmiums, and constructed a nanoparticle that trapped the amino-flavone. Next, to target the nanoparticle against tumor cells, a nanobody against the EGF receptor was attached to the nanoparticle. The EGF receptor is overexpressed on most cancer cells, and the anti-EGF receptor nanobody can direct the nanoparticle to the cancerous tissue [21, 22]. To study the effect of this structure on cancer cells, MDA-MB-468 (a TNBC cell line) was used to create a xenograft mouse model. After tumorigenesis in mice, the manufactured nanoparticle was administered to the mice, and due to the use of quantum dots in the nanoparticle structure, drug accumulation was tracked in the tested mice. The results showed that the designed nanoparticles accumulated in the tumor tissue without any systemic toxicity evaluated with organ histological analyses and body weight consideration. The nanoparticles also suppressed tumor growth in the xenograft mouse model [20].

The combination of nanobodies with another ligand or molecule has been used in many studies, and its benefits in the treatment of various diseases have been investigated. In a landmark study by Kitamura et al. [23], a combination of anti-EGFR nanobody and TNF-related apoptosis-inducing ligand (TRAIL) that binds to the DR4/5 receptor was used to treat breast cancer. In this project, the effect of each of these factors on breast cancer cells was investigated, and it was shown that anti-EGFR nanobody and death receptor ligand (DRL) alone have little apoptotic effect on cancer cells, but when these two molecules are joined together, they can effectively induce apoptosis in TNBC cells. To study the effect of this dual-functional EGFR and DR4/5-targeted on the brain metastatic of basal-like breast cancer, mesenchymal stem cells were created that were able to secrete this bi-functional molecule. These mesenchymal stem cells were used in mouse models of residual tumor after macro-metastasis resection, perivascular niche micro-metastasis, and leptomeningeal metastasis. The results showed that the secretion of the designed molecule by mesenchymal stem cells reduced the tumor volume and increased the survival of mice [23].

### Nanobodies against CD3

One of the most important ways to activate immune responses is to use anti-CD3 monoclonal antibodies. After binding of the T cell receptor (TCR) to a particular antigen, CD3, as a part of the TCR, is required for activation of T cells. Studies have shown that anti-CD3 antibodies are able to induce T cell proliferation and promote chemokine secretion in the absence of antigen interacting with T cells [24]. Consequently, anti-CD3 antibodies are used as an effective approach to activate T-cells, independent of TCR specificity [25]. In two separate experiments, a research team inserted the binding region of classical antibodies against the epsilon chain of CD3 into the nanobody structure by protein engineering and examined the new structure in activating immune cells against the mouse model of breast cancer. Initially, a nanobody against the epsilon chain of CD3 was fabricated by bioinformatics tools, and its binding was confirmed by docking. In the next step, the effect of this nanobody produced in a mouse model of breast cancer was investigated, and the results showed that administration of this nanobody increased the number of T cells (both CTLs and CD4 +) in the tumor environment, but the CD4 + / CD8 + T cell ratio decreased in the anti-CD3 nanobody receiving group. The administration of this nanobody also reduced the tumor volume in the recipient mice and increased their survival time [26]. In their second work, they showed that administration of nanobody against CD3 could reduce angiogenesis and tumor growth. This reduction in angiogenesis is due to a decrease in VEGFR2 expression and suppresses the expression of MMP9 in the tumor microenvironment [27].

### Nanobodies against CTLA-4

CTLA-4 is an important negative stimulatory agent in T cells, and the interaction of this molecule with B7 prevents further activation of T cells. CTLA-4 is expressed in some cancerous cells. The anticancer role of the CTLA-4 molecule makes it a perfect target in the treatment of tumors [28]. Tang et al. produced a nanobody against CTLA-4 to develop a way to inhibit cancer cells. The researchers first fused HepG2 and MCF-7 cancer cells with dendritic cells to stimulate T cells against the two cancer cell lines. The nanobody binds to CTLA-4 on the surface of T cells and stimulates the immune response against cancer cells. These stimulated T cells, in combination with two DC/HepG2-FCs or DC/MCF 7-FCs cells, can produce tumor-specific CD8 + cells. Tumor-specific CD8 + T cells generated in vitro were then tested on cancer cell cultures and used to eradicate HepG2 and MCF 7 tumors in NOD/SCID mice (in vivo). The results showed that the cells produced by this method reduced the tumor volume and increased the survival times of mice [29].

### Nanobodies against cell signaling proteins

STAT is a transcription factor, which shows expression in nearly 75% of human cancers, such as breast cancer [30]. Research shows that STAT-3 is overexpressed and activated in many cancers. It then goes to the cell nucleus and activates genes involved in the growth and differentiation of cancer cells. In order to eliminate breast cancer cells, Singh et al. [31] targeted STAT-3. In this study, human STAT-3 was injected into camels to isolate a nanobody with a suitable binding against this factor. The obtained nanobody was tested in different cells of human breast cancer lines such as MCF-7, BT474, and MDA-MB-231, -468, and -453 and in animal models of these cell lines. Results exhibited that this nanobody in cell culture was able to prevent the proliferation of TNBC cells and in the xenograft mode of these cancers to reduce tumor volume. Given that STAT3 is highly expressed in many malignant cancer cells, it may be possible to test the use of anti-STAT3 nanobody in the treatment of various cancers [31].

The most commonly over-activated pathway in malignancies is the PI3K-AKT pathway [32]. AKT (a serine/threonine protein kinase) is phosphorylated using two kinase enzymes, and this phosphorylation increases its stability [33]. The members of this pathway are susceptible to multiple genetic mutations, and a specific mutation in PI3K increases AKT activity and stability [34, 35]. Due to the role of AKT in cell proliferation, growth, migration, metabolism, and survival of cancer cells and the high incidence of this kinase in cancers (> 50%), AKT has become a valuable target in cancer diagnosis and treatment [36]. In the study by Merckaert et al., they showed that both AKT2 nanobodies (namely, Nb8 and Nb9) were able to moderate AKT2 and decrease the viability and proliferation of MDA-MB-231 cells. The Nb8 nanobody attached to the hydrophobic motif of AKT2 and inhibited IGF-1-induced phosphorylation. The expression and phosphorylation of proteins downstream of AKT were also influenced by this nanobody, which led to autophagy stimulation, G0/G1 cell cycle arrest, loss of stress fibers, and a decrease in the number of the focal adhesion molecules. They mapped a part of the AKT2 pathway by means of a specific nanobody and confirmed that AKT2 and its hydrophobic motif could be considered as potential targets for cancer treatment. Nanobodies were chosen because they remain functional when expressed in the reducing environment of cytoplasm in mammalian cells, allowing them to modulate endogenous proteins in a relevant context [37].

Studies have shown that Ras-associated RHO-GTPase are major regulators of cell signaling pathways, which play a critical role in cell division, invasion, and migration processes. Due to the role of RHOA subfamily members (RHOA, RHOB, and RHOC) in tumorigenesis and metastatic dissemination, these proteins have been extensively studied [38–40]. In many malignancies, such as colon and breast cancer, the expression of members of the RHOA family is out of control, although the correlation between the expression level of these proteins and tumor invasion is still unclear [39, 40]. Keller et al. [38] established a nanobody-based ELISA test with enhanced selectivity, which permitted the reliable quantification and detection of RHO protein GTP-bound in the nM range. This method is highly effective in both cell lines and tumor samples. They performed an acceptable analysis of the active state of RHOA-like and RAC1 in tumor samples, with the most complete examination of RHOC-GTP and RHOA-GTP levels in human breast tumor specimens. They demonstrated increased activity of GTP-bound RHOC and RHOA in tumors compared to natural tissues, and showed that RHO expression and RHO activation are two independent parameters in the different types of breast cancer [38].

### Nanobodies against extracellular matrix and cytoskeleton proteins

ECM is a major component of the tumor microenvironment and plays an important role in cancer cell survival, metastasis, and angiogenesis [41]. ECM is regenerated in the progression of various diseases, including cancer, and therefore contains proteins that are found specifically and abundantly in the disease area. As a result, ECM proteins are a promising target for the development of cancer therapy and diagnostic methods [42, 43]. A nanobody, NJB2, was created by Jailkhan and coworkers [44] against an alternatively spliced region of fibronectin that is expressed in cancerous ECM and neovascular. In a range of breast cancer models, including mouse and human TNBC as well as melanoma, they used NJB2 nanobody to detect primary tumors and metastatic areas with great specificity. The NJB2 nanobody was employed for imaging of pancreatic ductal adenocarcinoma (PDAC) in a mouse model. With superior S/N ratios than conventional 2-fluorodeoxyglucose PET/CT imaging, the NJB2 identified PDAC tumors as well as early pancreatic lesions. Moreover, NJB2 was used to detect pulmonary fibrosis in an experimental model. They recommended NJB2 and other anti-ECM nanobodies as prospective candidates for nanobody-based therapeutic applications and for early non-invasive diagnostics of malignancies and metastatic lesions [44].

Abnormal circulation of the actin cytoskeleton is thoroughly associated with the invasion and migration of cancer cells. Proteomic studies have shown that one of the components of the actin cytoskeleton, called CapG, is overexpressed in breast malignancy. Previous studies propose that CapG is involved in the proliferation and metastasis of tumor cells and therefore can be considered as one of the drug targets in breast cancer [45, 46]. Impe et al. [47] investigated the potential therapeutic effect of blocking CapG activity by using llama-derived anti-CapG-specific nanobodies in a mouse model of breast cancer metastasis. They produced nanobodies against human CapG and used them as intrabodies in breast cancer cells. They used orthotopic and tail vein in vivo models of metastasis in nude mice to investigate the spread of cancer cells. Using F-actin and G-actin binding experiments, they discovered a nanobody against CapG that binds to the first CapG domain with a nanomolar affinity. Thus, the interaction of CapG with actin monomers or filaments was inhibited. Moreover, intracellular delocalization tests revealed that the nanobody could interact with CapG in the cellular cytoplasm. Cell migration and Matrigel invasion were inhibited by the expression of the nanobodies in breast cancer cells. It is important to note that the nanobody prevented the lung metastatic lesions' formation in a xenograft mouse model. In their experiments, they also successfully delivered CapG nanobodies into MDA-MB-231 cells using non-pathogenic bacteria harboring a type III protein secretion system [47].

### Nanobodies against carbonic anhydrase IX (CAIX)

Aram and colleagues [48] developed a specific carbonic anhydrase IX nanobody for molecular imaging of pre-invasive breast malignancy. In this study, the carbonic anhydrase was chosen to obtain high tumor-specificity, because it is possible to achieve higher tumor-specificity by directly targeting proteins that are upregulated under hypoxic conditions, such as carbonic anhydrase IX (CAIX). CAIX expression is regulated by hypoxia inducible factor 1α (HIF-1α), a transcription factor that is stabilized under hypoxic conditions. This nanobody was conjugated with IRDye800CW. The CAIX-specific nanobodies were site-specifically conjugated to IRDye800CW after selection by a modified phage display technique. The effects of the nanobodies on xenograft transplanted breast cancer mouse models were evaluated by means of DCIS. Anti CAIX nanobody injection to DCIS xenografts mice with CAIX overexpression demonstrated a TNR of 4.3 compared to the negative control group with a TNR of 1.4 after 2 h. In addition, a TNR of 1.8 was achieved for DCIS mice. Moreover, Biodistribution investigation showed an uptake of 14.0% I.D./g and 4.6%I.D./g in DCIS + CAIX tumors and DCIS tumors groups respectively. However, this parameter was obtained at about 2.0%I.D./g for negative control. These results indicate that successful production of an IRDye800CW conjugated anti-CAIX nanobody can be used as a rapid imaging approach for pre-invasive breast cancer [48].

## Discussion

TNBC tumor heterogeneity, as a reason for the different clinical consequences of this malignancy, has led to different responses to traditional therapies. As a result, it often leads to different survival times [49]. However, due to the advance of molecular mechanisms of cancer, TNBC, which was previously considered an unattainable disease by molecular therapy, has recently attracted the attention of researchers for new targeted therapies [49]. Antibodies can inhibit tumors in a variety of ways by binding specifically to their targets. On the other hand, antibody–drug conjugates have caused a great deal of excitement in the specific treatment of tumors [50]. Given the problems of conventional antibodies, this article attempts to introduce nanobodies in the diagnosis of TNBC. Different studies show that the use of nanobodies to detect tumor antigens is more sensitive to antigen diagnosis than conventional antibodies. The longer CDR domains in VHHs lead to better interaction with antigens [21, 51]. Research shows that for the effective treatment of cancer, several therapeutic factors must be used simultaneously. For example, combining chemotherapy with immunotherapy is much more effective than treatment with either of these methods alone. According to the results collected in this study, in order to have a more effective treatment method, it is better to use a combination of several nanobodies, each of which identifies a different target. The advantage of this method is that this combination simultaneously activates several different pathways to kill cancer cells and reduces the risk of cancer cells resisting treatment. Simultaneous use of several nanobodies in this method can be in the form of separate administration of nanobodies or in the form of developing diabody, tri-body, etc., consisting of several nanobodies with different properties. These cocktails of different nanobodies can effectively kill TNBC tumor cells by combining them with the traditional method of treatment (chemotherapy). We propose to use multi-subunit toxins in combination with different nanobodies to effectively treat this cancer, so that each of these toxin subunits is attached to a specific nanobody against specific antigens important in TNBC. This causes the toxin to be activated only in TNBC cancer cells with all the targets and specifically causes the death of cancer cells. To better detect TNBC tumor cells, it also used this method and attached a specific marker to each specific nanobody to make the diagnosis more precise and specific.

However, nanobodies have some disadvantages, including rapid renal clearance and induced nephrotoxicity [52]. In the diagnosis application of nanobodies, these disadvantages are not troublesome, but these defects can be problematic in therapeutic application. However, numerous approaches have been established to improve the half-life of nanobodies and decrease renal clearance, including the construction of dia-, tri-, or tetra-body fragments by conjugating specific nanobodies against the target antigen or designing and developing nanobodies with a high affinity to human albumin [8]. Another way to improve the half-life of nanobodies is to bind them to the Fc of human antibodies or attach the nanobodies to polyethylene glycol [25]. Moreover, protein engineering was applied to increase the specificity and potency of existing VHHs. In this way, molecules with good specificity and efficiency against TNBC antigens can be achieved from existing nanobodies, so that these new nanobodies can detect new epitopes [32]. Finally, it is with great optimism that we look to future trials that increase our understanding of the molecular mechanisms of TNBC and pave the way for increasing the number of specific targets and enhanced outcomes for patients. Moreover, considering the specificity and efficiency of VHHs, it seems the use of VHHs will be more widespread in the treatment and detection of cancers in the near future. Table 1 summarizes the nanobodies against “triple negative breast cancer” antigens that can be used in the diagnosis and treatment of TNBC.

**Table 1:** List of Nanobodies against TNBC antigens

Name	Type of antigen	Sample	Binding affinity (Kd)	Refs.
Anti-TNF-VHH	TNFα	Cell line	2.05 nM	[2]
RGD4C- VHH	TNFα	Cell line, mouse model	0.1062 nM	[1]
7D12 Nb	EGFR	Cell line, mouse model	5 nM	[20]
E_V_DR_L_	EGFR	Mouse model	0.5 µM	[23]
Anti-CD3 epsilon nanobody	CD3	Mouse model	N.A	[26]
Anti-CD3 epsilon nanobody	CD3	Mouse model	N.A	[27]
CTLA-4 Nb16	CTLA-4	Cell line	N.A	[29]
Anti-STAT3 B VHH13	STAT3	Cell line, mouse model	22 nM	[31]
Nb8 Nb9	AKT2	Cell line	118 nM 0.5 nM	[37]
RH12	RHO protein	Tissue	1.52 nM	[38]
NJB2	FN-EIIIB in the ECM	Mouse model	2.25 nM	[44]
CAPNb2	CapG	Cell line	23 nM	[47]
CAIX1 Nb CAIX4 Nb	CAIX	Cell line, mouse model	6 nM 2 nM	[48]

## Conclusion

Nanobodies or VHHs have good properties such as small size, facile and low-cost production, and high stability. These advantages make them attractive as a cancer therapeutic or diagnostic agent. These nanobodies produced against antigens expressed in TNBC such as TNF-α, EGFR, CD3, CTLA-4, STAT3, AKT2 and other antigens can be used for the purpose of detecting, targeting, or sensing cancerous cells. 

## Data Availability

Not applicable.

## Abbreviations

TNBC: Triple-negative breast cancer
HCAbs: Camel heavy chain antibodies
FRs: Framework regions
EMT: Epithelial mesenchymal transition
TRAIL: TNF-related apoptosis-inducing ligand
DRL: Death receptor ligand
TCR: T cell receptor
STAT: Signal transducer and activator of transcription
ECM: Extracellular matrix
PDAC: Pancreatic ductal adenocarcinoma
S/N: Signal to noise
DCIS: Ductal carcinoma in situ cells
TNR: Tumor-to-normal tissue ratio
I.D./g: Injected dose per gram of tissue
